# On the Binormal Predictive Receiver Operating Characteristic Curve for the Joint Assessment of Positive and Negative Predictive Values

**DOI:** 10.3390/e22060593

**Published:** 2020-05-26

**Authors:** Gareth Hughes

**Affiliations:** SRUC, Scotland’s Rural College, The King’s Buildings, Edinburgh EH9 3JG, UK; gareth.hughes@sruc.ac.uk

**Keywords:** diagnostic test, evaluation, ROC curve, PROC curve, binormal, prevalence, positive predictive value, negative predictive value, Bayes’ rule, leaf plot, expected mutual information

## Abstract

The predictive receiver operating characteristic (PROC) curve is a diagrammatic format with application in the statistical evaluation of probabilistic disease forecasts. The PROC curve differs from the more well-known receiver operating characteristic (ROC) curve in that it provides a basis for evaluation using metrics defined conditionally on the outcome of the forecast rather than metrics defined conditionally on the actual disease status. Starting from the binormal ROC curve formulation, an overview of some previously published binormal PROC curves is presented in order to place the PROC curve in the context of other methods used in statistical evaluation of probabilistic disease forecasts based on the analysis of predictive values; in particular, the index of separation (PSEP) and the leaf plot. An information theoretic perspective on evaluation is also outlined. Five straightforward recommendations are made with a view to aiding understanding and interpretation of the sometimes-complex patterns generated by PROC curve analysis. The PROC curve and related analyses augment the perspective provided by traditional ROC curve analysis. Here, the binormal ROC model provides the exemplar for investigation of the PROC curve, but potential application extends to analysis based on other distributional models as well as to empirical analysis.

## 1. Introduction

The predictive receiver operating characteristic (PROC) curve is a diagrammatic format introduced by Shiu and Gatsonis [1] in the context of the statistical evaluation of probabilistic disease forecasts. Such forecasts are often evaluated by calculation of metrics defined conditionally on the actual disease status. Metrics defined conditionally on the outcome of the forecast—predictive values—are also important, although less frequently reported; this motivates the introduction of the PROC curve. Although this approach is potentially useful, as yet it has not been commonly applied [2]. One possible reason for this is the apparent complexity of patterns generated by PROC curve analysis [1,3]. Thus Shiu and Gatsonis note that “It is therefore essential to study and attempt to characterize the geometric properties of PROC curves before undertaking an investigation of how the curves can be used to evaluate the performance of a diagnostic test”.

This article is intended as a contribution towards furthering an understanding of some of the properties of PROC curves as described by Shiu and Gatsonis [1]; and thus, hopefully, increasing applications of their analysis. The approach taken is to place the PROC curve in the context of some other methods for the statistical evaluation of probabilistic disease forecasts. In particular, we discuss the receiver operating characteristic (ROC) curve [4,5], the index of separation PSEP [6], and the leaf plot [7,8], in terms of their relationship to the PROC curve. The article is set out as follows. Section 2 provides background to the methods discussed as context for PROC curve analysis. Section 3 presents an analysis of some particular PROC curves, and the perspective provided by analyses based on corresponding contextual methods. Section 4 is a concluding general discussion. 

## 2. Methods

The preliminary steps leading towards the calculation and analysis of a PROC curve largely follow the route mapped by Sackett et al. [4], particularly Chunk #2 and Chunk #3 of Chapter 4 on the interpretation of diagnostic data. The obvious difference is that the impetus of Sackett et al. is data-driven, whereas here the required observations are represented by normal distributions. As in ROC analysis, it is not necessary for test data to follow normal distributions. Here, the normality assumption is helpful in the investigation of the theoretical properties of the curve and the exploration of scenarios that lead to different shapes. To begin, we consider two groups of subjects for which the known actual (‘gold standard’) status is denominated case ‘*c*’ or non-case ‘*nc*’. For each subject, a second observation is available, referred to generically as a risk score. The risk score may be useful as a proxy variable in diagnosis when obtaining the gold standard at the outset may be considered too time-consuming, difficult, or expensive; or when an early estimate of risk may facilitate preventative treatment. By convention, the risk score is calibrated so that *c* subjects tend to have higher scores than *nc* subjects, although typically there is overlap between the ranges of scores for the two groups.

Now consider a threshold on the risk score scale, such that a score above the threshold (designated ‘+’) is taken as indicative of likely *c* status, while a score at or below the threshold (designated ‘−‘) is taken as indicative of likely *nc* status. The resulting two-way classification of subjects provides the basis for a 2 × 2 prediction-realization table, which may be based on numerical data as in Table 4-3 of Sackett et al. [4] or on probabilities as in the present analysis (Table 1). From Table 1, with *i* = +, − (for the predictions) and *j* = *c*, *nc* (for the realizations), we write *p_i_*_∩ *j*_ = *p_j_*_∩ *i*_ = *p_i_*_|*j*_ ∙ *p_j_* = *p_j_*_|*i*_ ∙ *p_i_* via Bayes’ rule. The *p_j_* are taken as the Bayesian prior probabilities of case (*j* = *c*, ‘prevalence’) or non-case (*j* = *nc*) status, such that *p_nc_* = 1 − *p_c_*. We can write *p_i_* = *p_i_*_|*c*_ ∙ *p_c_* + *p_i_*_|*nc*_ ∙ *p_nc_* (*i* = + or −) via the Law of Total Probability.

The conditional probability *p*_+|*c*_ is referred to as the true positive proportion (TPP, sometimes *sensitivity*). The conditional probability *p*_−|*nc*_ is referred to as the true negative proportion (TNP, sometimes *specificity*). We refer to the conditional probability *p*_+|*nc*_ that is the complement of specificity as the false positive proportion (FPP = 1 − TNP). TPP and TNP are metrics often used in the evaluation of tests based on 2 × 2 tables. TPP characterizes the proportion of *c* subjects that had + test outcomes, while TNP characterizes the proportion of *nc* subjects that had – test outcomes. TPP and TNP are metrics defined conditionally on actual disease status, independent of prevalence.

Returning to the matter of the threshold on the risk score scale, consider now the problem of placement of this threshold. The effect of different threshold placements can be investigated by generating a set of 2 × 2 tables derived from a sequence of thresholds on the risk score scale, calculating the TPP and TNP values from each table, and then plotting the graph of TPP against FPP (= 1 − TNP). This graph is the receiver operating characteristic (ROC) curve, see [4,5]. Generally, risk score threshold values increase along an ROC curve from lower values in the top right-hand corner to higher values in the bottom left-hand corner. When both the case and non-case distributions of risk scores are modeled as normal distributions, we have a binormal ROC curve, a format that has been extensively studied, see for example Section 4.4 of [5].

The conditional probability *p_c_*_|+_ is referred to as the positive predictive value (PPV). This characterizes the posterior probability of *c* status given a + test outcome. The conditional probability *p_nc_*_|−_ is referred to as the negative predictive value (NPV). This characterizes the posterior probability of *nc* status given a − test outcome. The metrics PPV and NPV are also applicable to the evaluation of tests based on 2 × 2 tables, but they are less frequently reported than TPP and TNP. At least in part, this is likely because PPV and NPV vary with prevalence. The effect of prevalence on predictive values was illustrated diagrammatically by Sackett et al., see Figures 4−9 and 4−10 of [4], and more recently by Coulthard [7] and by Coulthard and Coulthard [8] as the leaf plot. This diagram shows how the posterior probabilities PPV and 1−NPV vary with prevalence, given the TPP and TNP values of the test in question. In effect, the diagram provides a nomogram for calculating probability revisions resulting from use of a test, via application of Bayes’ rule.

## 3. Results

### 3.1. Binormal ROC Curves

Section 3 of [1] is devoted to an exploration of properties of the theoretical PROC curve, and in particular to a detailed investigation of the shape properties of the PROC curve arising from the binormal ROC model. The results presented here begin with identification of characteristics of the binormal ROC curves corresponding to some qualitatively different PROC curves presented in [1] (Table 2). As in [1], the binormal ROC model is written so that the non-case distribution is standard normal (see Table 2).

**Figure 1:**
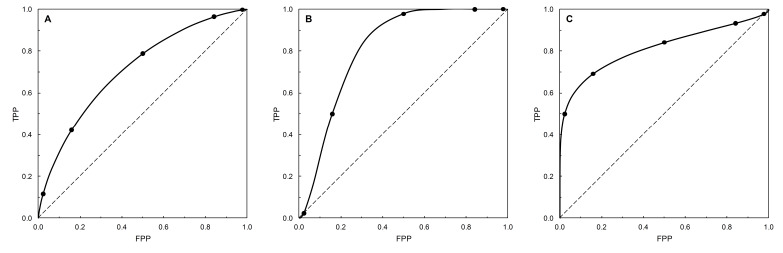
ROC curves based on data from Table 2. The curves are calibrated at intervals of the risk score threshold; starting in the top right-hand corner, −2, −1, 0, 1, 2. (**A**) Examples 1 and 2 (they have identical ROC curves), Symmetric. (**B**) Example 3, TNP-asymmetric, t* = 0.02275, s* = 2. (**C**) Example 4, TPP-asymmetric, t* = 0.97725, s* = −2.

From Table 2, we write *a* = (*μ_c_* − *μ_nc_*)/*σ_c_* and *b* = *σ_nc_*/*σ_c_*, then the binormal ROC curve is TPP = *f*(FPP) = Φ(*a*+*b*∙Φ^−1^(FPP)) [5] in which Φ denotes the standard normal cumulative distribution function and Φ^−1^ its inverse (Figure 1). Visually, the resulting ROC curves appear to be either symmetric about the negative diagonal of the graphical plot (Figure 1A), or skewed towards the upper axis (referred to as TNP-asymmetry, Figure 1B) or the left-hand axis (referred to as TPP-asymmetry, Figure 1C) of the plot. More formally, the shape (symmetry) properties of ROC curves have been characterized in terms of the Kullback-Leibler divergences *D*(*f_c_*‖*f_nc_*) and *D*(*f_nc_*‖*f_c_*) between the case and non-case distributions [9,10,11] (with *D*(*f_c_*‖*f_nc_*) and *D*(*f_nc_*‖*f_c_*) ≥ 0, and equality only if the case and non-case distributions are identical). In particular, binormal ROC curves may be symmetric, TNP-asymmetric or TPP-asymmetric [10]; for symmetric binormal ROC curves, *D*(*f_c_*‖*f_nc_*) = *D*(*f_nc_*‖*f_c_*); while for TNP-asymmetric binormal ROC curves, *D*(*f_c_*‖*f_nc_*) < *D*(*f_nc_*‖*f_c_*), and for TPP-asymmetric binormal ROC curves, *D*(*f_c_*‖*f_nc_*) > *D*(*f_nc_*‖*f_c_*). For the binormal ROC curve in particular, these conditions reduce to symmetry when *σ_c_* = *σ_nc_*, TNP-asymmetry when *σ_c_* < *σ_nc_*, and TPP-asymmetry when *σ_c_* > *σ_nc_* [10] (Table 2, Figure 1).

Note that the main diagonal of an ROC plot, where TPP = FPP, is indicative of a situation where the distributions of risk scores for cases and non-cases are identical. A risk score threshold *s* anywhere on this line is thus characteristic of a test that provides no discrimination between cases and controls (a situation which, of course, is undesirable). Looking again at Figure 1, the ROC curves depicted appear either to fall entirely above the main diagonal of the plot (Figure 1A), or to cross it (Figure 1B,C). ROC curves of the former type are referred to as ‘proper’, and the latter type as ‘improper’ [12] (where the part of the curve under the diagonal is sometimes referred to as a ‘hook’). More formally, the above-noted symmetry conditions also incorporate the conditions for which binormal ROC curves are either proper or improper. Symmetric curves are proper (*σ_c_* = *σ_nc_*, *b* = 1), TNP-asymmetric curves are improper (*σ_c_* < *σ_nc_*, *b* > 1) and cross the main diagonal of the plot from below with increasing FPP (Figure 1B), and TPP-asymmetric curves are improper (*σ_c_* > *σ_nc_*, *b* < 1) and cross the main diagonal of the plot from above with increasing FPP (Figure 1C). From [12], for improper curves we can calculate *ρ* = (*μ_c_* − *μ_nc_*)/(*σ_c_* − *σ_nc_*), and then t* = Φ(*ρ*) is the value of FPP where the ROC curve crosses the main diagonal and s* = −*ρ* is the risk score threshold on the curve at the point where it crosses the diagonal (Figure 1).

### 3.2. The Corresponding PROC Curves

**Figure 2:**
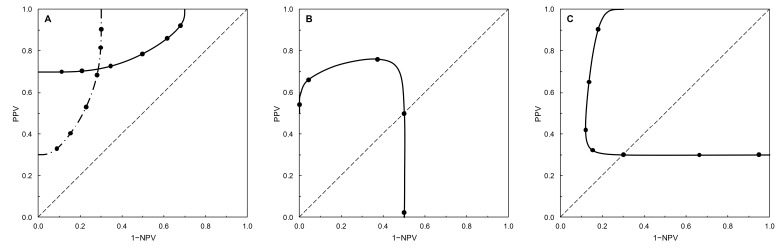
PROC curves based on data from Table 2. The curves are calibrated at intervals of the risk score threshold. (**A**) Example 1 (solid line, prevalence = 0.7; calibration points starting from the left-hand vertical axis are −3, −2, −1, 0, 1, 2) and Example 2 (dot-dash line, prevalence = 0.3; calibration points starting from the left-hand vertical axis are −1, 0, 1, 2, 3, 4). These examples have identical ROC curves (see Figure 1) but different PROC curves because the prevalence differs. (**B**) Example 3, prevalence = 0.5, calibration points starting from the left-hand vertical axis are −1, 0, 1, s* = 2, 3. (**C**) Example 4, prevalence = 0.3, calibration points starting from the right-hand vertical upright are −4, −3, s* = −2, −1, 0, 1, 2.

Figure 2 shows the PROC curves corresponding to the binormal ROC curves shown in Figure 1. These graphs appear in Figures 1 and 2 of [1] (see Table 2), minus the main diagonal and the calibration. The correspondence is as follows. Take a point on an ROC curve (as characterized by a particular risk score threshold value) in Figure 1 and note the matching TPP and FPP values. Now, given a value for the prevalence, we can calculate the corresponding PPV and 1−NPV values via Bayes’ rule. These values then define a point on the corresponding PROC curve in Figure 2 that is characterized by the same risk score threshold value as the point on the ROC curve from which we started. Thus we can denote the risk score threshold on a PROC curve at the point where it crosses the diagonal using the same notation (s*) as for an ROC curve.

On the main diagonal of the ROC plot, where TPP = FPP, we find via Bayes’ rule PPV = 1 − NPV = *p_c_* (the prior probability, estimated by prevalence). In words: if a test provides no discrimination between cases and non-cases, its application leaves the posterior probabilities unchanged from the prior. Typically, test evaluation on the basis of an ROC curve is concentrated on regions where the curve is above the main diagonal (i.e., TPP > FPP), because via Bayes’ rule this implies PPV > *p_c_* and NPV > *p_nc_*.

Figure 2A shows two PROC curves based on the same ROC curve (Figure 1A), the difference resulting from different prevalence values. The ROC curve is symmetric (proper, *b* = 1); the corresponding PROC curves are monotone [1]. In each case, PPV and 1−NPV increase as the risk score threshold increases (i.e., there is a trade-off between PPV and NPV along the PROC curve), and the curves do not cross the main diagonal of the plot.

Figure 2B,C show PROC curves based on improper (*b* ≠ 1) ROC curves (Figure 1B,C, respectively). The PROC curves are non-monotone [1] and cross the main diagonal of the plot. In Figure 2B the PROC curve is based on a TNP-asymmetric ROC curve crossing the diagonal from below as FPP increases, the crossover point (s* = 2) being in the bottom left-hand corner of the ROC plot. In Figure 2C the PROC curve is based on a TPP-asymmetric ROC curve crossing the main diagonal of the plot from above as FPP increases, the crossover point (s* = −2) being in the top right-hand corner of the ROC plot. Although the PROC curves’ shapes are very different to the shapes of the corresponding ROC curves, interpretation is aided if we note that in each case the same range of risk scores is of interest. In Figure 1B and Figure 2B, risk scores < 2 fall above the main diagonal of the plots. In Figure 1C and Figure 2C risk scores > −2 fall above the diagonals. In each case, these are the ranges of risk scores that would typically be considered as possible test thresholds. The part of the PROC curves in Figure 2B,C below the main diagonal of the plots, corresponding to the improper ROC ‘hooks’ in Figure 1B,C, appears more pronounced, but covers the same range of risk score thresholds in each case.

With these interpretations of PROC curves in relation to their corresponding ROC curves, we may investigate the properties of a putative test by first setting a risk score threshold based on designated TPP and FPP values, as is typical current practice. If we then locate this threshold value as a point on the corresponding PROC curve, we can trace from this point to the horizontal and vertical axes of the PROC plot to establish the x,y coordinates of the point, and so obtain the corresponding predictive values at that threshold. Thus ROC curves and PROC curves in combination may contribute to test evaluation.

### 3.3. Measures of Predictive Performance

**Figure 3:**
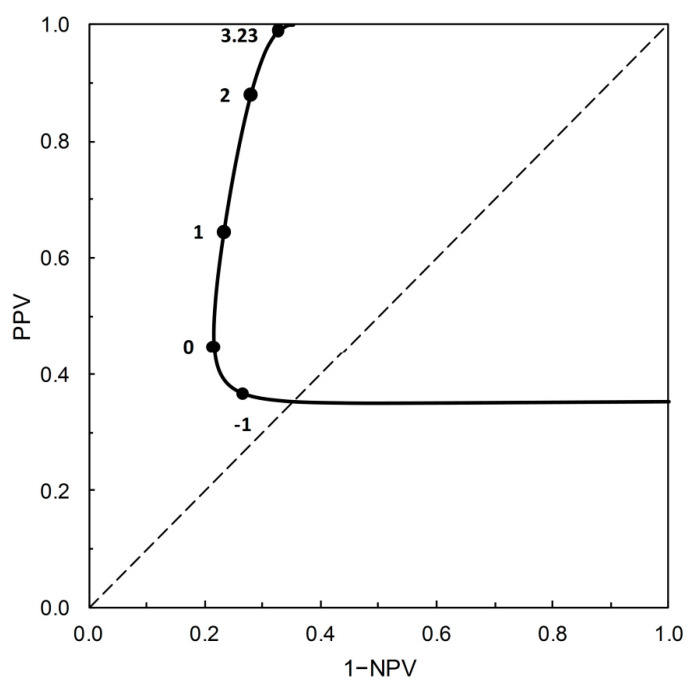
The PROC curve based on Example 5 (Table 2), with prevalence = 0.35 [13]. The curve is calibrated at intervals of the risk score threshold as it increases above the main diagonal at points −1, 0, 1, 2, and *s_opt_* = 3.23 (at which PPV = 0.990, 1 − NPV = 0.326).

**Figure 4:**
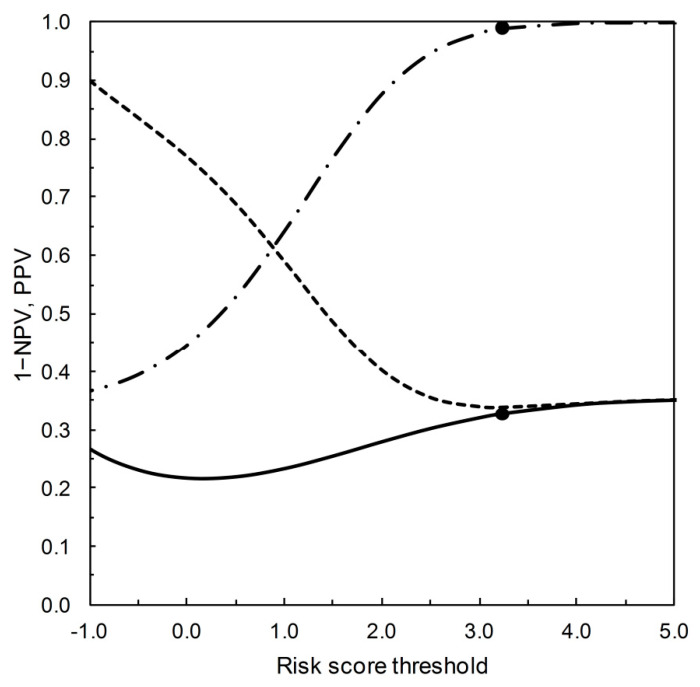
PPV and 1−NPV vary with the risk score threshold as characterized by the PROC curve in Figure 3. Only risk score thresholds that correspond to the part of the PROC curve above the main diagonal of the plot (as calibrated in Figure 3) are shown here. The PPV (dot-dash line) and 1−NPV (solid line) traces characterize the predictive performance measures PSEP = PPV − (1−NPV) and *r* = (1 − PPV) + (1 − NPV). The *r* trace (dashed line) reaches a minimum value of *r_opt_* = (1 − 0.990) + (1 − 0.674) = 0.336 at risk score threshold *s_opt_* = 3.23 [1]. The markers (●) on the predictive value traces at risk score threshold = 3.23 (PPV = 0.990, 1 − NPV = 0.326) indicate where this graphical plot coincides with Table 3.

Figure 3 shows the PROC curve corresponding to the Example 5 from Table 2. This example provides a context for Shiu and Gatsonis [1] to address the question of how to evaluate predictive performance based on the PROC curve. Here, we approach this problem by replotting the data on which Figure 3 is based in an alternative format. Figure 4 shows predictive values PPV and 1−NPV on the vertical axis, with the risk score threshold on the horizontal axis. Only risk score thresholds that fall above the main diagonal of the PROC plot shown in Figure 3 are included in Figure 4.

Figure 4 embodies two potential measures of predictive performance, the index of separation PSEP [6] and the distance to perfect prediction *r* [1]. PSEP provides a measure of ‘prognostic information’ in the situation where a test is applied to a validation data set, PSEP = PPV − (1−NPV). Note that PSEP is a probability measure [6], and not based on information in the Shannonian sense (see [14] for further discussion). In Figure 4, PSEP is the distance between the PPV and 1−NPV traces, varying with risk threshold score. Now suppose we start from the concept of a notionally perfect predictions, for which PPV = NPV = 1. Shiu and Gatsonis [1] define the distance from a given test to notional perfection as *r* = (1−PPV) + (1−NPV), varying with risk score threshold. In Figure 4, *r* is the sum of the distances from the PPV trace to 1 and from the 1−NPV trace to 0. Thus we have *r* = 1−PSEP.

Shiu and Gatsonis [1] interpret the minimum value of *r* (denoted here *r_opt_*) as the notional best achievable test performance from a given PROC curve, and *s_opt_* as the corresponding optimal risk score threshold. However, as Shiu and Gatsonis [1] point out, there may be practical restrictions that militate against the operational use of this optimal threshold. The variation in predictive values in the neighborhood of the threshold identified as optimal is thus likely to be of interest in the process of selecting a value for operational use.

**Figure 5:**
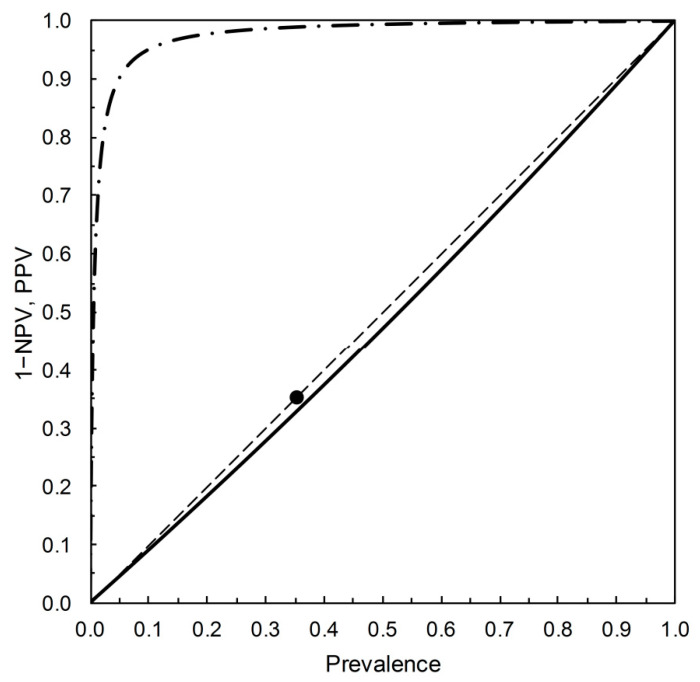
The leaf plot based on Example 5 (Table 2). The PPV trace (dot-dash line) shows that a + test outcome at almost any prevalence provides a useful indication of case status. However, the 1−NPV trace (solid line) shows that a − test outcome is of little value in ruling out non-case status. The marker (●) on the prevalence trace (dashed line) at 0.35 (where PPV = 0.990, 1 − NPV = 0.326) indicates where this graphical plot coincides with Table 3.

Let us compare Figure 4 with the corresponding leaf plot [7,8]. To do so, we require values of TPP and TNP for the test characterized by the risk score threshold value of *s_opt_* = 3.23 on the PROC curve for Example 5 (Table 2). Using the data for Example 5 as given in Table 2, we can calculate the ROC curve (not shown). We may then refer to the risk score threshold value of 3.23 on this ROC curve and so obtain the corresponding values of TPP and FPP as 0.109 and 0.001 respectively (so TNP = 1 − FPP = 0.999). These are the data required for completion of Table 3. The table is calculated as follows: *p*_+∩*c*_ = TPP∙*p_c_*, *p*_+∩ *nc*_ = FPP∙(1 − *p_c_*), *p*_−∩ *c*_ = (1 − TPP)∙*p_c_*, *p*_−∩ *nc*_ = (1 − FPP)∙(1 − *p_c_*), results shown to 2dp. The resulting leaf plot is then shown in Figure 5. From the leaf plot, we see that PPV and 1−NPV increase as prevalence increases. The leaf plot (Figure 5) embodies the predictive performance measures PSEP and *r* in the same way as Figure 4. The vertical distance between the leaf margins at any given prevalence is a PSEP value, and *r* = 1 − PSEP.

On the vertical (probability scale) axis of both Figure 4 and Figure 5 are PPV and 1−NPV. The two diagrams differ in terms of what drives variation in these predictive values. In Figure 4, the prevalence is constant and PPV and 1−NPV vary as TPP and FPP (and thus the risk score threshold) vary. In Figure 5 (the leaf plot), TPP and FPP (and thus the risk score threshold) are constant and PPV and 1−NPV vary as the prevalence varies. The numerical version of the 2 × 2 prediction-realization table (Table 3) for the test characterized by the risk score threshold value of *s_opt_* = 3.23 on the PROC curve (Figure 3) describes the point at which Figure 4 and Figure 5 coincide. 

### 3.4. An Information Theoretic Perspective on Predictive Performance

The predictive performance metrics *r* and PSEP for PROC curves are measured on a probability scale; each provides a description of separation between prior and posterior probabilities which varies with prevalence. The corresponding approach to describing the performance of tests by means of ROC curves has been discussed in detail by Pepe, see Section 4.3 of [5]. In essence, an ROC curve provides a description of the separation between the distributions of risk scores for cases and non-cases. Indices that summarize ROC curves thus provide a summary of the separation between these two distributions. Separation between the distributions of risk scores is independent of prevalence, so we require this also of our summary indices. The most commonly-used such summary index is the area under the ROC curve.

If we wish instead to evaluate performance by measuring distances on an information theoretic scale, we must similarly distinguish between separation between prior and posterior probabilities (which depends on prevalence) and separation between (summaries of) distributions of risk scores (which does not). In both cases we may calculate relative entropies (Kullback-Leibler distances) in order to characterize separation. On the one hand, relative entropy calculations may be used to describe distances between distributions of risk scores [15,16], on the other, to describe distances between prior and posterior probabilities [17]; the details differ in each case as outlined in [18].

As applied in the present context, relative entropies are metrics that quantify diagnostic information from + and from – test outcomes (as expectations over both actual states). That is to say, diagnostic information is quantified in terms of prior and posterior probabilities. Then expected relative entropy, with the expectation calculated over both test outcomes, is equal to expected mutual information. From Table 3, we can directly calculate expected mutual information, *I*, via:

$$I=\sum_{i=+,-}\;\sum_{j=c,nc}p_{i\;\cap \;j}\cdot \ln\left[\frac{p_{i\;\cap \;j}}{p_{i}\cdot p_{j}}\right]$$

from which, on substituting the numerical data from the table, we obtain $\hat{I}=0.04$ nats. This value corresponds to the risk score threshold *s_opt_* = 3.23 on Figure 3 and Figure 4. If we estimate *I* for further thresholds along the PROC curve shown in Figure 3, the resulting values form a curve with maximum value of $\hat{I}\approx 0.09$ nats at a risk threshold 1 < *s* < 2. In this way, expected mutual information can provide an information theoretic perspective on the evaluation of predictive performance for a PROC curve. For further discussion of the qualitative correspondence between PSEP (on a probability scale) and *I* (on an information scale) as measures of separation, see [14].

## 4. Discussion

Shiu and Gatsonis [1] provide a comprehensive introduction to the predictive receiver operating characteristic curve for the joint assessment of positive and negative predictive values and its potential application in the evaluation of diagnostic tests. To realize its applicability, the PROC curve needs to become part of what Gatsonis [19] has called ‘ROC thinking’—whereby the operational risk score threshold value is considered not just in relation to TPP and FPP (defined conditionally of the actual disease status) but also in relation to predictive values (defined conditionally on the outcome of the forecast). The PROC curve is part of such thinking. The PROC curve provides a format for test evaluation that is focused on predictive values and, in addition, links to the index of separation PSEP [6] and the leaf plot [7,8].

Because the PROC curve often displays complex patterns arising from the way that changes in the risk score threshold affect predictive values, interpreting a PROC curve requires both attention to detail and appropriate contextual data. The following recommendations are based on investigation of the PROC curves as characterized in Table 2, based on binormal ROC curves.

- The PROC curve and the ROC curve should not be regarded as mutually exclusive formats. Both perspectives contribute to test evaluation. In addition, use of both perspectives also serves a pedagogic function. The ROC curve is estimated conditionally on disease status whereas the PROC curve is estimated conditionally on test result. With both frames of reference available for consideration, carefully distinguishing between them becomes a requisite component of the presentation of a test’s evaluation statistics.
- Generally (and especially in the case where the background distributional model for case and non-case risk scores may lead to an improper ROC curve) it is useful to include the main diagonal on the graphical plot of the PROC curve. It is also useful to present the full zero-one range of the axes of the plot, even if (e.g., in the case of an empirical analysis) the PROC curve data do not extend over the full range. Standardizing our view of the PROC curve helps in interpretation and comparison of the apparently complex patterns generated by PROC curve analysis.
- Calibrating a PROC curve at intervals with values of the risk score threshold is useful, because the way that the threshold changes along PROC curves is less obviously straightforward than for ROC curves.
- It is always useful to include details of the prevalence value for which a PROC curve has been calculated along with the graphical plot. Availability of a prevalence value allows further calculations (via Bayes’ rule) following selection of a test based on a particular risk score threshold on the PROC curve.
- If a test is characterized on the basis of a threshold on the PROC curve, it is then useful to gain a wider perspective by determining TPP (sensitivity) and TNP (specificity) and calculating the leaf plot (for which purpose a spreadsheet is freely available [8]).

PROC curve analysis draws particular attention to the importance of predictive values in the evaluation process, and so in turn brings the index of separation PSEP [6] and the leaf plot [7,8] within the scope of Gatsonis’ [15] ‘ROC thinking’. Here we have considered PROC curves based on binormal ROC curves; other distributional models are available. When the background distributional models for case and non-case risk scores are continuous, there is a common information theoretic basis for understanding the shape properties of ROC curves [11] that can also contribute to our interpretation of the corresponding PROC curves. There is also an information theoretic approach to the assessment of predictive performance based on PROC curves.

The suggestions presented here are aimed at furthering adoption of the PROC curve and related methods for assessment of predictive values in the context of diagnostic test evaluation. As a set of methods they usefully augment the perspective provided by traditional ROC curve analysis used in isolation. Here, the binormal ROC model has provided the exemplar, but analysis based on risk score frequencies described by other distributional models would be of interest, as would empirical analysis (see, e.g., [20,21,22]). As yet there are few disease-related PROC analyses in the literature [2]; we expect this to change as understanding of the contribution that the format can make to test evaluation grows.

## Figures and Tables

**Table 1:** The prediction-realization table for a test with two categories of realized (actual) status (*c*, *nc*) and two categories of prediction (+, −). In the body of the table are the joint probabilities.

**Prediction (*i*)**	**Realization (*j*)**		
	*c*	*nc*	Row sums
+	*p*_+ ∩ *c*_	*p*_+ ∩ *nc*_	*p*_+_
−	*p*_− ∩ *c*_	*p*_− ∩ *nc*_	*p*_−_
Column sums	*p_c_*	*p_nc_*	1

**Table 2:** Example data and binormal ROC curve terminology.

		Case (*f_c_*)		Non-case (*f_nc_*)				
Example	Source	*μ_c_* ^i^	*σ_c_* ^ii^	*μ_nc_* ^i^	*σ_nc_* ^ii^	ROC Curve Symmetry ^iii^	ROC Curve Proper or Improper ^iv^	ROC Curve Crosses Diagonal ^v^
1.	[1] Figure 1*b* ^vi^	0.8	1	0	1	Symmetric	Proper	N/A
2.	[1] Figure 1*b* ^vii^	0.8	1	0	1	Symmetric	Proper	N/A
3.	[1] Figure 2*a*	1	0.5	0	1	TNP-asymmetric	Improper	From below
4.	[1] Figure 2*b*	2	2	0	1	TPP-asymmetric	Improper	From above
5.	[1] Figure 6	1.134	1.704	0	1	TPP-asymmetric	Improper	From above

^i^ Mean; ^ii^ Standard deviation; ^iii^ Terminology of [10]; ^iv^ Terminology of [12]; ^v^ As FPP increases; ^vi^ Prevalence = 0.7; ^vii^ Prevalence = 0.3.

**Table 3:** The numerical prediction-realization table for a test with two categories of realized (actual) status (*c*, *nc*) and two categories of prediction (+, −) based on Example 5 (Table 2).

**Prediction (*i*)**	**Realization (*j*)**		
	*c*	*nc*	Row sums
+	*p*_+ ∩ *c*_ = 0.04	*p*_+ ∩ *nc*_ = 0.00	*p*_+_ = 0.04
−	*p*_− ∩ *c*_ = 0.31	*p*_− ∩ *nc*_ = 0.65	*p*_−_ = 0.96
Column sums	*p_c_* = 0.35	*p_nc_* = 0.65	1
